# Causal Relationship between Immune Cells and Gynecological Cancers through Bidirectional and Multivariable Mendelian Randomization Analyses

**DOI:** 10.7150/jca.92627

**Published:** 2024-02-04

**Authors:** Yangyang Zhang, Yangyuxiao Lu, Xuanyu Wang, Keren He, Mengqi Fang, Jiabao Xu, Ye Xu, Fangfang Tao, Ping Lü

**Affiliations:** 1 Institute of Obstetrics and Gynecology, Hospital of Obstetrics and Gynecology, Fudan University, Shanghai, China; Shanghai Medical College, Fudan University, Shanghai, China.; 2 The First Clinical Medical College, Zhejiang Chinese Medicine University, Hangzhou, Zhejiang, China.; 3 College of Traditional Chinese Medicine, Tianjin University of Traditional Chinese Medicine, Tianjin, China.; 4 Department of Immunology and Microbiology, Basic Medical College, Zhejiang Chinese Medical University, Hangzhou, Zhejiang, China.; 5 Department of TCM, Taizhou First People's Hospital, Taizhou, Zhejiang 318020, China.

**Keywords:** immune, gynecological malignancies, breast cancer, Mendelian randomization

## Abstract

**Background:** Evidence suggests potential associations between gynecological malignancies and various immune cell chemicals and systems. However, establishing a causal relationship remains uncertain.

**Methods:** This work employed Wald ratio for one single-nucleotide polymorphism (SNP) or the inverse-variance weighted method (IVW) for multiple SNPs to conduct bidirectional two-sample Mendelian randomization (MR) analysis by utilizing genome-wide association study (GWAS) data. We employed supplementary methods, including MR-Egger and weighted median methods, to detect and correct for the influence of horizontal pleiotropy. In addition, we also use colocalization analysis for further validation.

**Results:** In IVW analysis, increases in relative count of circulating CD11c^+^ HLA-DR^++^ conventional dendritic cells (cDC) were associated with an elevated risk of breast cancer (OR [95% CI], 1.1295 [1.0632-1.2000], P = 8.044 × 10^-5^), while elevated levels of HLA-DR on plasmacytoid dendritic cells (DC) and HLA-DR on DC were protective against breast cancer. In addition, actual count of CD39^+^ resting Treg AC was also shown to be causally associated with the development of ovarian cancer, whereas a high relative count of CD28^+^ CD45RA^-^ CD8^+^ T cells reduced the risk of cervical cancer. Sensitivity analysis revealed almost no evidence of bias in the current study. Multivariable MR (MVMR) analyses further confirmed a direct impact of the CD11c^+^ HLA-DR^++^ cDC immune phenotype on breast cancer. Colocalization analysis showed the lead SNP, rs780094, suggesting HLA-DR GWAS shared a common genetic mechanism with breast cancer.

**Conclusions:** The MR study identified significant causal relationships between multiple immunophenotypes and breast cancer, aiming to provide clinicians with some reference for cancer prediction and explore further potential associations between immune phenotypes and gynecologic tumors.

## 1. Introduction

Gynecologic neoplasms represent a substantial public health concern, affecting a significant portion of the population. The American Cancer Society recently released statistics showing that breast cancer (BRCA) is the most frequent cancer in women worldwide and accounts for one-third of all malignancies diagnosed in women. The incidence rate of breast cancer has recently seen a massive rise, posing an escalating burden on global health systems^1^. Breast cancer currently ranks as the most prevalent malignancy among Chinese women, constituting 12.2% of all new global breast cancer diagnoses and accounting for 9.6% of worldwide breast cancer-related mortalities^2^. Relevant research has demonstrated the high degree of malignancy, poor prognosis, late recurrence, and metastasis associated with breast cancer^3^, particularly metastatic breast cancer, for which the five-year survival rate is only 26%^4^. Ovarian cancer (OC)^5^ and cervical cancer^6^ are also leading causes of death in gynecologic cancer patients.

The foundation of clinical treatment for gynecologic cancer remains chemotherapy, which could keep the survival rate of patients at a high level^6^-^8^. Although chemotherapeutic medications have a therapeutic function, patients will quickly develop drug resistance. Despite the fact that it is now known that the FRAT1 protein^3^, BRCA1/2^9^, and vitamin D receptor (VDR)^10^ are linked to a poor prognosis for breast cancer, early detection of the disease is still challenging, and there are currently no reliable objective biological predictors^11^. OC's intractability is significantly increased because it is typically identified at a late stage and has often expanded outside the ovaries^12^.

Gynecologic neoplasms are an enormous concern and are present in a large number of people, as evidenced by the fact that the incidence of cancer is rising annually in women while falling in males^13^. 731 immune cell characteristics were found at 70 loci, shedding light on the chemicals and processes underlying cellular control^14^. Our study of immune cells having a potential relationship with gynecologic tumors may provide new ideas for early diagnosis.

Chimeric antigen receptor (CAR) T-cell therapies, commonly utilizing autologous CAR-T cells to produce targeted antibodies against cancer, have gained significant traction as a burgeoning immunotherapeutic approach for breast cancer in recent years.^15^. Several dendritic cells (DC) -specific vaccinations to boost immunity provide novel^16^, targeted therapies for the illness, and immunotherapy is anticipated to raise the survival rate of breast cancer^17^.

Mendelian randomization (MR)is a technique that employs genetic variation as an instrumental variable (IV) to explain the degree of the causal influence of exposure on a risk factor^18^. Reverse MR has the advantage of eliminating the effects of reverse causality bias and confounding^19^. The benefit of Multivariable MR (MVMR) analysis is that it allows for the simultaneous consideration of the effects of several variables. This helps reduce confounding bias and improve comprehension of the interactions between various variables and their individual and combined impacts on the experimental results^20^. It hasn't yet been used to look at connections between immunological roles and gynecologic neoplasms. We conducted a two-sample MR analysis with the most recent genome-wide association study (GWAS) pooled data to thoroughly evaluate the association between immune cells and gynecologic malignancy risk and anticipate specific indicators beforehand.

## 2. Materials and Methods

### 2.1 Study Design

Two-sample MR was used to evaluate the relationship between 731 immune cells and gynecological tumors, including breast cancer, ovarian cancer, and cervical carcinoma. Our study followed the prescribed guidelines of the Strengthening the Reporting of Observational Studies in Epidemiology-Mendelian Randomization (STROBE-MR) checklist^21^. Since MR analysis uses genetic variation to represent risk factors, valid IVs in causal inference must meet three essential presumptions: (1) it is directly linked to exposure; (2) it is unrelated to potential confounders between exposure and result; and (3) it does not affect the outcome through mechanisms other than exposure. Most of the summary statistics utilized in the MR came from earlier research projects, while individual and ethical approval was obtained for all original studies.

### 2.2 GWAS Data Sources for Immune Traits

The GWAS Catalog provides public GWAS summary information for every immune trait (accession numbers from GCST90001391 to GCST90002121)^14^. It contains a total of 731 immunophenotypes, consisting of absolute cell (AC) counts (n=118), median fluorescence intensities (MFI) reflecting surface antigen levels (n=389), morphological parameters (MP) (n=32) and relative cell (RC) counts (n=192), which was collected from 3,757 European individuals, with no overlapping subjects among the cohorts^22^. Following adjustment for confounding variables such as sex and age, the study investigated the associations among approximately 22 million single-nucleotide polymorphisms (SNPs) that were genotyped using high-density arrays and imputed utilizing a Sardinian sequence-based reference panel^23^. Specifically, the GWAS on 272 blood immune-cell-related traits was conducted in 1,629 individuals from the Sardinian population. Subsequent GWAS analyses of up to 1,000 individuals identified 28 additional loci associated with immune cell traits. Additionally, 539 immune traits were profiled using flow cytometry^14^.

### 2.3 Oncology GWAS Data Sources

**Figure 1:**
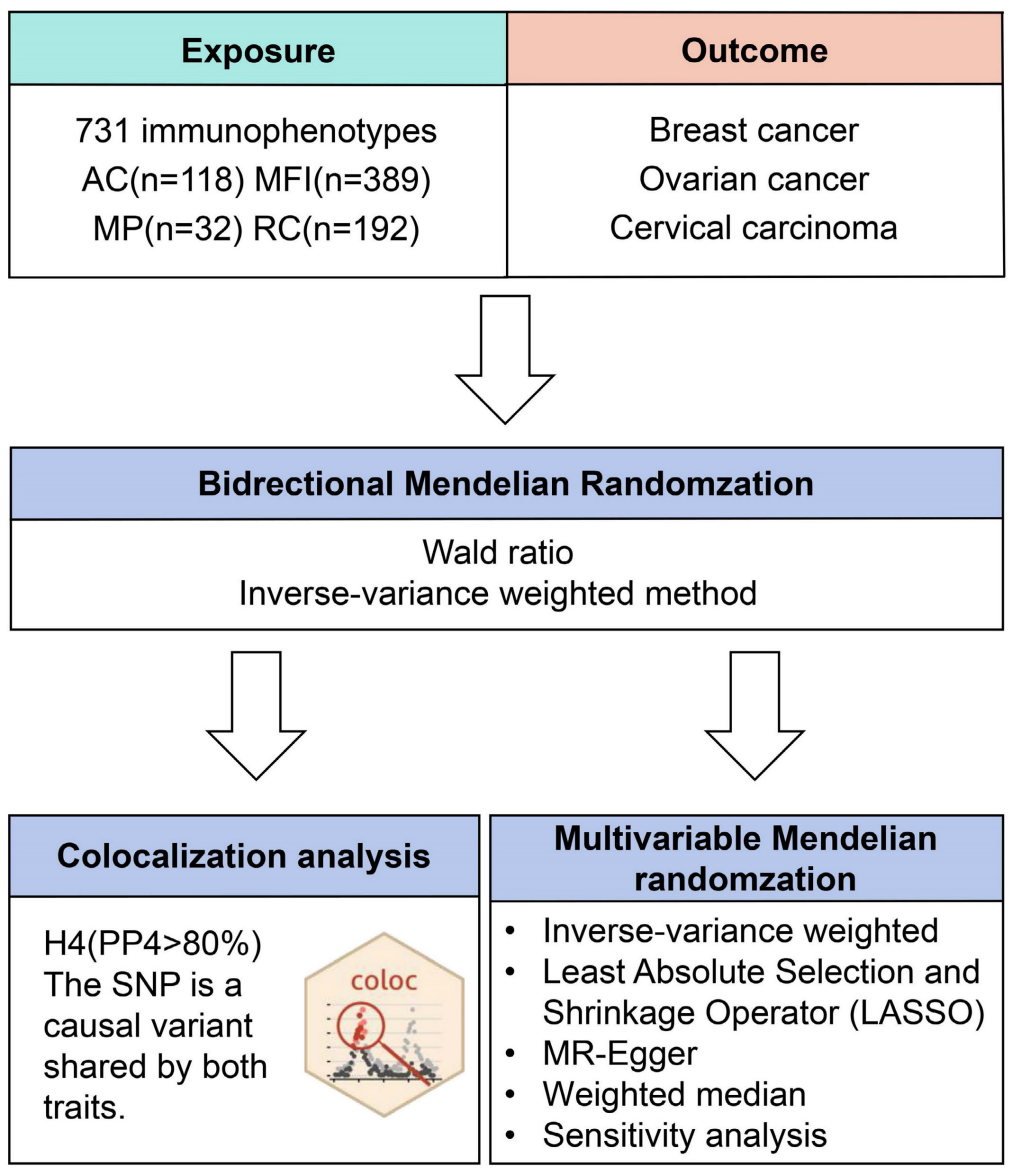
Flow diagram of study design. AC: absolute cell. MFI: median fluorescence intensities. MP: morphological parameters. RC: relative cell.

Figure 1 illustrates our study design. Initially, data from the FinnGen database (R9 data, https://www.finngen.fi/fi)^24^. The data on breast cancer involved females of European origin with a mean age of 58.66 years at the first incident (15,680 cases, 167,189 controls), yielding an unadjusted disease rate of 7.44%. Additionally, there were 168,214 cases of ovarian cancer with a mean age of 58.38 for the first event (1,025 cases, 167,189 controls) (Table S3). As for the GWAS data of cervical carcinoma, there were 1,167,637 (2,236 cases, 165,401 controls) women whose age at first detection of the disease was 37.85 years old (Table S3). The inclusion and exclusion criteria for patient data on gynecological cancer are based on ICD-10.

### 2.4 Instrumental Variables

Owing to the small number of SNPs found, we ensured that SNPs are strongly associated with immunological features by setting the IVs' statistical significance level threshold to P < 5 × 10^-6^ for each immune trait^25^. Second, we set the linkage imbalance (LD) R^2^ threshold at 0.001 within a 1000 kb distance to produce independent IVs^26^, ^27^. To guarantee the validity of the reference chain, we then eliminated palindrome SNPs with moderate allele frequency (MAF) and MAF less than 0.01^28^. F-statistics were computed to quantify sample overlap effects and weak instrument bias, considering the rather loose threshold. A sufficient degree of association between SNP and phenotype is indicated by F-statistics larger than 10^29^. We have ultimately located 8926 IVs following the screening above. Finally, we used PhenoScanner to identify disease-related SNPs and eliminate confounding factors that may impact results 30.

### 2.5 Mendelian Randomization Analyses

This study conducted all analyses using two-sample MR and Mendelian Randomization R Package. Our primary analysis technique is the Random Effects Inverse Variance Weighted (IVW) model, which could yield precise estimations if all contained SNPs are used as valid IVs^31^. We use MR-Egger regression, IVW analysis, and Cochran's Q test to evaluate the heterogeneity of instrument performance and prevent the IV hypothesis of MR from being broken^32^. When P < 0.05, heterogeneity is considered to exist. At the same time, MR-Egger regression and MR-PRESSO global test can be used to evaluate horizontal pleiotropy^31^, ^33^. The above results are presented through leave-one-out analysis, scatter plots, and funnel plots. Reverse MR was used to estimate the effect of outcome on exposure to demonstrate the reverse causality between them^34^.

### 2.6 Multivariable Mendelian Randomization Analysis

Controlling the overlap process between exposure factors is necessary to identify important exposure factors independent of other factors and assess their direct impact on the findings. Therefore, we used IVW, MR-Egger, least absolute selection and shrinkage operator (LASSO), and weighted median methods to evaluate the exposure strength. MR-Egger regression analysis can detect pleiotropy, while its intercept regression can detect potential horizontal pleiotropy^35^. To rule out heterogeneity effects, the IVW and MR-Egger methods assess whether specific variable features significantly affect the connection between the independent and dependent variables^36^. Lasso regression is used to screen for risk factors^37^.

### 2.7 Colocalization Analysis

To evaluate the potential overlap of genetic variants between critical immune cells and breast cancer, we conducted Bayesian colocalization analyses utilizing the coloc R package. Additionally, we employed the LocusCompareR R package to represent the colocalization outcomes visually colocalization. To ensure comprehensive coverage, we expanded the genomic region surrounding the lead SNPs to 100 kb in both directions. In this study, we assumed that there could be a maximum of one association per trait in the test region. We employed approximate Bayesian factorization to determine the likelihood of different configurations between the two traits. This allowed us to calculate posterior probabilities (PPs) for five possible hypotheses: (1) H0, which suggests no association with either trait; (2) H1, indicating association with trait 1 but not trait 2; (3) H2, suggesting association with trait 2 but not trait 1; (4) H3, indicating association with both trait 1 and trait 2, with two independent SNPs; and (5) H4, suggesting association with both trait 1 and trait 2, with one common SNP. Each configuration's designated PPs are PP0, PP1, PP2, PP3, and PP4. Noteworthy PP4s (e.g., PP4 > 80%) are robust evidence for colocalization, indicating a shared variant between these immune cells and breast cancer.

## 3. Results

### 3.1 Exploration of the Causal Effect of Immunophenotypes on Gynecological Tumors

To estimate the causal effect of immunophenotypes on breast cancer, in the two-sample MR analysis, IVW was the primary method of calculation (Table S6). We adopted a multiple-testing-adjusted threshold of P < 6.84 × 10^-5^ (0.05/731) using the Bonferroni correction to declare a statistically significant, causal relationship 38. After adjusting for multiple tests, we found three effective positive results in breast cancer.

**Figure 2:**
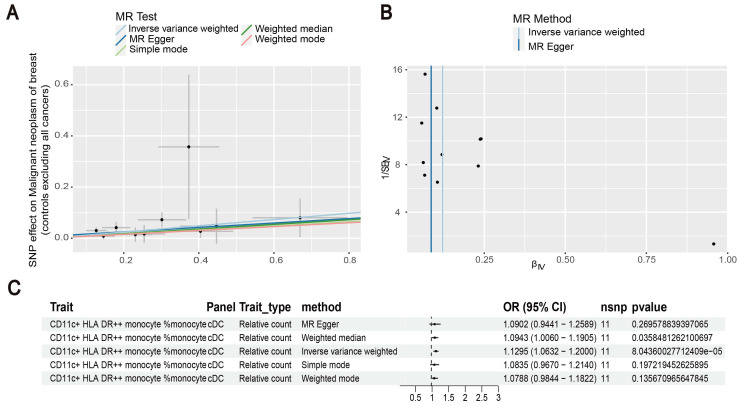
Exploration of the causal relationship between CD11c^+^ HLA-DR^++^ monocyte and breast cancer. (A) Increased levels of CD11c^+^ HLA-DR^++^ monocyte led to the development of ovarian cancer. (B) Inverse-variance weighted (IVW) and MR-Egger for the link between CD11c^+^ HLA-DR^++^ monocyte and breast cancer. (C) Exploring the causal relationship between CD11c^+^ HLA-DR^++^ monocyte and breast cancer.

**Figure 3:**
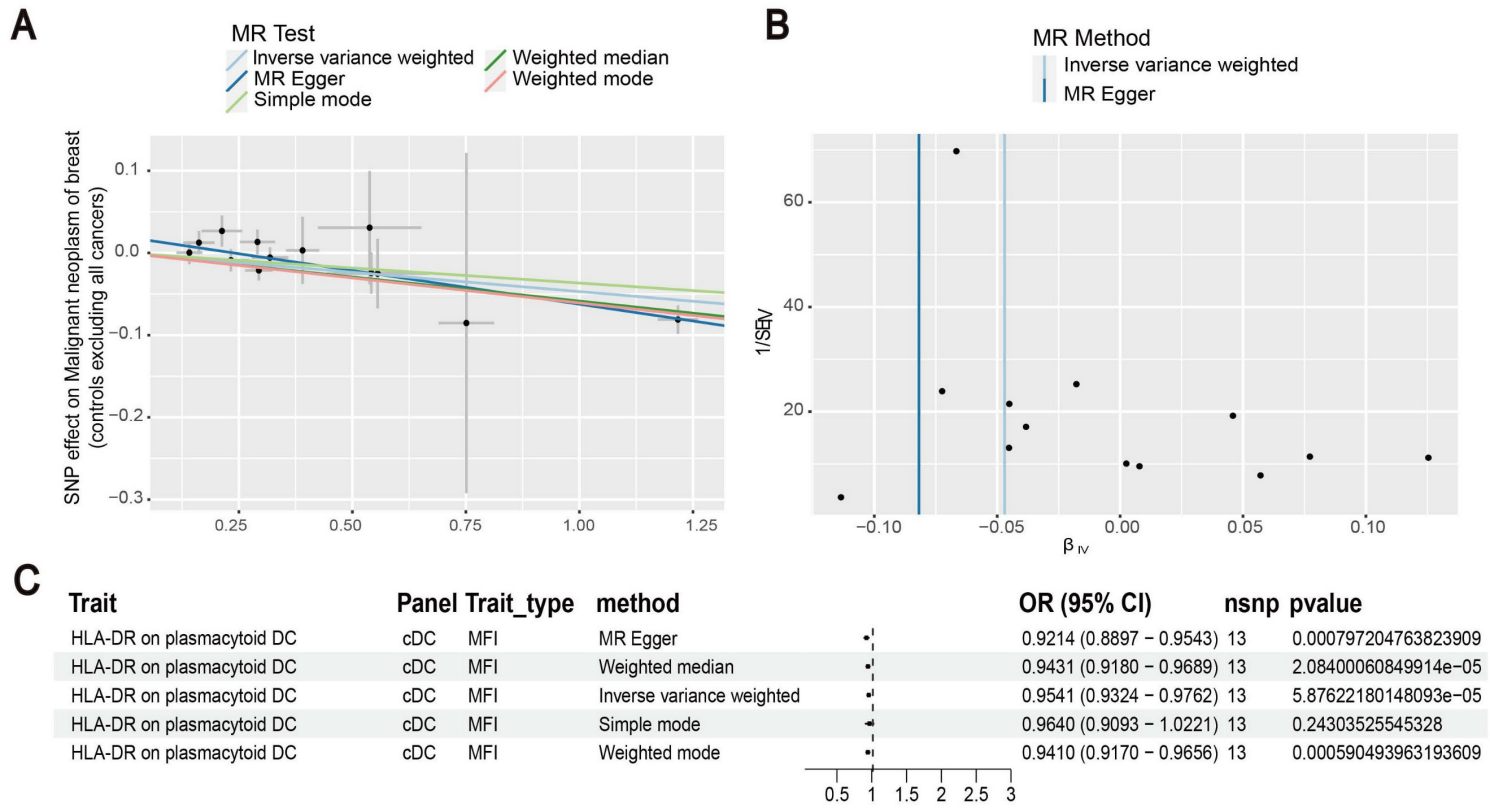
Estimating the causal effects of HLA-DR on plasmacytoid DC on breast cancer. (A) Decreased levels of HLA-DR on plasmacytoid DC caused the risk of breast cancer. (B) The scatter plot showed the association between HLA-DR on plasmacytoid DC and breast cancer. (C) Illustration of the relationship between HLA-DR on plasmacytoid DC and breast cancer by several Mendelian randomization (MR) analysis methods.

**Figure 4:**
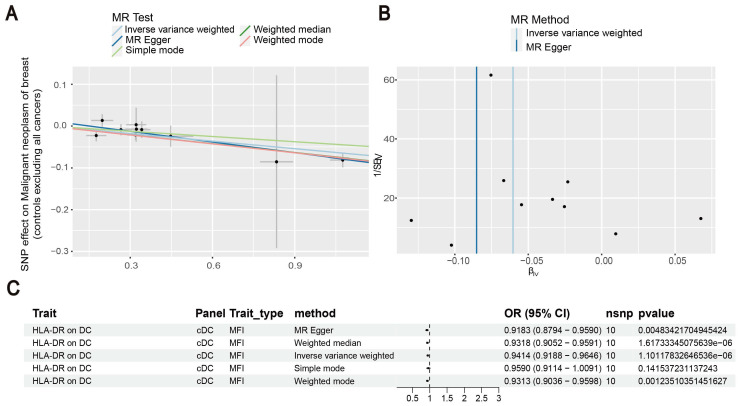
Investigating the causal relationship between HLA-DR on DC and breast cancer. (A) Decreased levels of HLA-DR on DC resulted in the development of breast cancer. (B) IVW and MR-Egger were used to estimate the causal effects. (C) The forest plot showed the link between HLA-DR on DC and breast cancer.

Our results showed that the odds ratio (OR) of CD11c^+^ HLA-DR^++^ conventional dendritic cells (cDC) on breast cancer development was estimated to be 1.1295 (OR [95% CI], 1.1295 [1.0632-1.2000], P = 8.044 × 10^-5^) (Figure 2). The strong causal relationship between HLA-DR on plasmacytoid DC and breast cancer was found in our results (OR [95% CI], 0.9541 [0.9324-0.9762], P = 5.876 × 10^-5^) (Figure 3). An increase in HLA-DR on DC led to decreased breast cancer incidence rates (OR [95% CI], 0.9414, [0.9188-0.9646], P= 1.101 × 10^-6^) (Figure 4). In addition, MR leave-one-out sensitivity analysis performed on the positive results indicated that the results were reliable.

**Figure 5:**
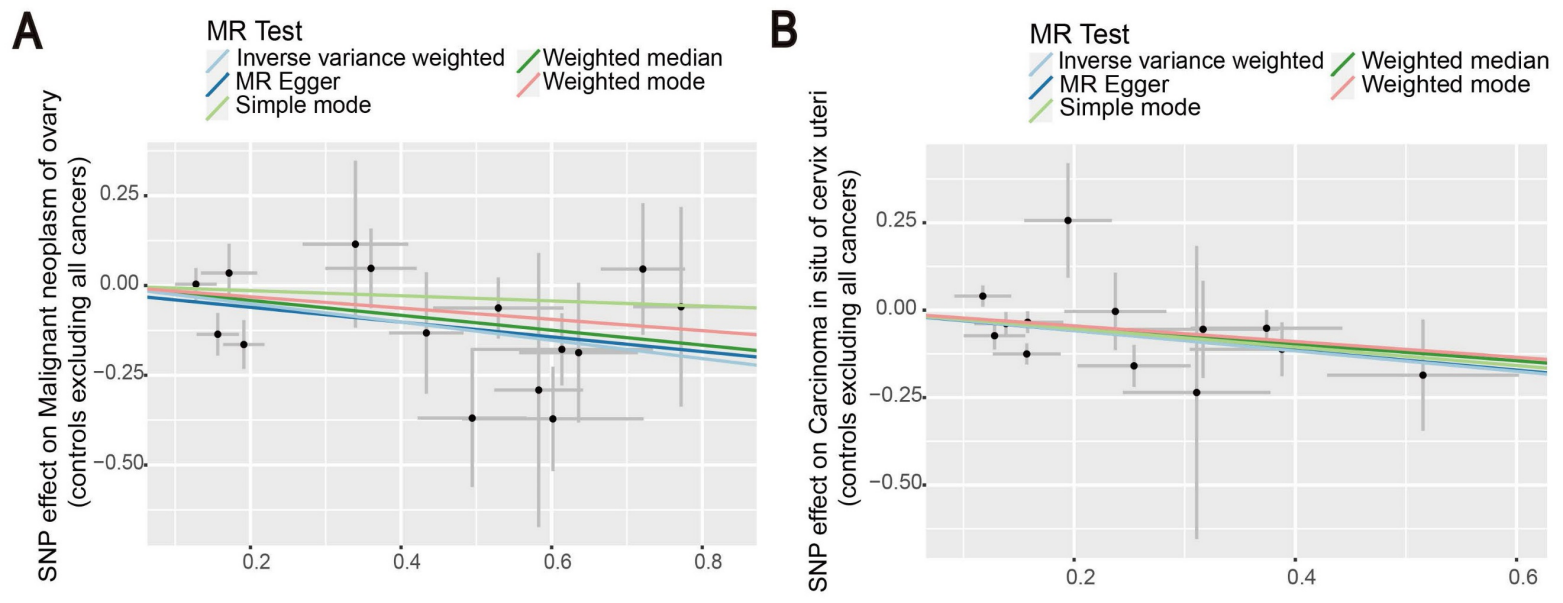
The scatter plots showed the causal relationship between immune traits in ovarian cancer and cervical cancer. (A) MR analysis showed the link between CD39^+^ resting Treg AC and ovarian cancer. (B) IVW, MR-Egger, Simple mode, Weighted median, and Weighted mode were used to investigate the relationship between CD28^+^ CD45RA^-^ CD8^+^T cells and cervical cancer.

Then, we proceeded to perform a two-sample MR analysis of immunophenotypes of ovarian cancer, and cervical carcinoma in situ using the IVW method (Table S4, S5). A wide variety of immune cells are shown in the volcano diagram in Figure S1. After Bonferroni correction, we found the causal effect of immunophenotypes on ovarian and cervical carcinoma in situ was attenuated, which did not reach a significant causality of 0.05. At the nominal significance level, two immunophenotypes with the smallest p-value were selected as results. Figure 5 shows that increased levels of CD39^+^ resting Treg AC reduce the incidence of ovarian cancer. Similarly, a negative correlation exists between CD28^+^ CD45RA^-^ CD8^+^ T cells and the development of cervical cancer, suggesting that the immunophenotype protects against cervical cancer (Figure 5).

Additionally, MR leave-one-out sensitivity analysis for CD28^+^ CD45RA^-^ CD8^+^ T cell and CD39^+^ resting Treg AC illustrated that the results are reliable. The funnel plot also proved our results again (Figure S2).

**Figure 6:**
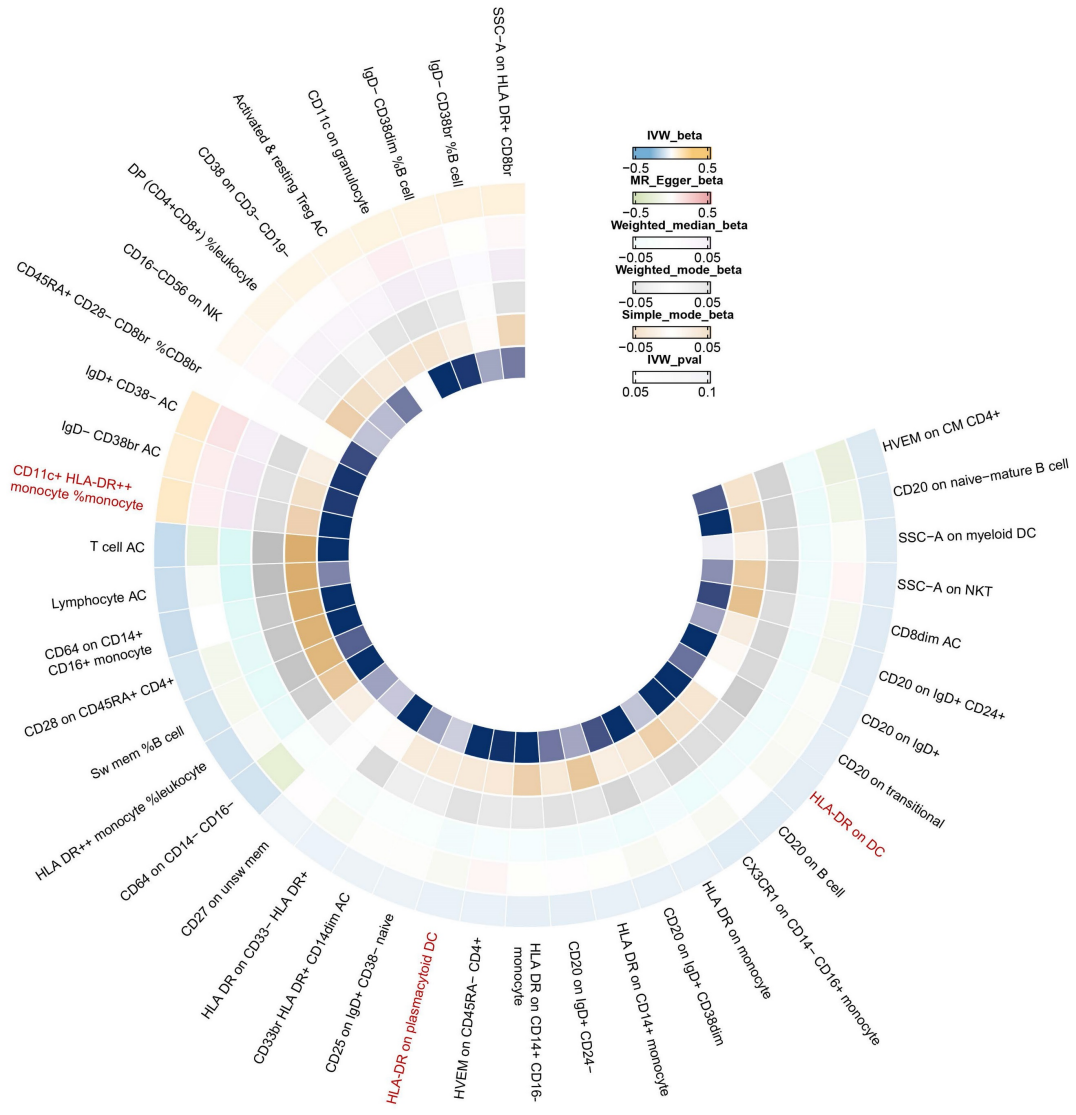
IVW Mendelian randomization illustrated the association between 731 immunophenotypes and breast cancer.

In Figure 6, we found significant positive results of three immunophenotypes CD11c^+^ HLA-DR^++^ cDC, HLA-DR on plasmacytoid DC, and HLA-DR on DC.

### 3.2 Exploration of the Causal Effect of Breast Cancer on Immunophenotypes

To further confirm the causal relationship between immunophenotypes and breast cancer, we performed reverse MR analyses for the three positive results mentioned above (CD11c^+^ HLA-DR^++^ cDC, HLA-DR on plasmacytoid DC, and HLA-DR on DC) and the results did not find any evidence of breast cancer's effect on the evidence of an impact of immunophenotypes risk. The IVW analyses demonstrated unidirectional causation with breast cancer, with p-values > 0.05 (Table S7), indicating that the tumor is not the cause of the immunophenotypic abnormalities.

### 3.3 Further Assessment of the Impact of Immunophenotypes on Breast Cancer Using MVMR

A univariate Mendelian randomization study confirmed a causal association between the three positive results of immunophenotypes (CD11c^+^ HLA-DR^++^ cDC, HLA-DR on plasmacytoid DC, and HLA-DR on DC) and the development of breast cancer, and that there was no significant effect of reverse MR analysis. Therefore, we designed a multivariable MR analysis to assess the impact of the above-risk immunogenic factors on breast cancer.

The results of multivariable MR analysis are shown in the Table S9. After correcting for other immunophenotypic traits using IVW, MR-Egger, Lasso, and weighted median, we found that the causal association between CD11c^+^ HLA-DR^++^ cDC and breast cancer remained statistically significant (P < 0.05). IVW results showed that increasing levels of the immunophenotype HLA-DR on plasmacytoid DC increased the incidence of breast cancer (P = 3.92 × 10^-5^), which was consistent with the results analyzed by the other three methods. In addition, significant causal associations disappeared after correction for MR analyses of two immunophenotypes, HLA-DR on plasmacytoid DC and HLA-DR on DC.

The intercept of MR-Egger (P = 0.08) suggests that there is no horizontal pleiotropy. In the multivariable MR study of MR-Egger and IVW methods, p-values > 0.05 showed no heterogeneity (Table S9).

### 3.4 Investigation of Potential Shared Genetic Variations between Immune Cells and Breast Cancer

Evidence supporting the presence of shared causal variants was obtained through the execution of colocalization analyses, which examined the association between three immune cell types (namely, HLA-DR on plasmacytoid dendritic cells, HLA-DR on dendritic cells, and CD11c^+^ HLA-DR^++^ monocyte) and breast cancer.

The results of our study reveal a statistically significant colocalization of shared genetic variants between breast cancer and HLA-DR on plasmacytoid dendritic cells, as well as between breast cancer and HLA-DR on dendritic cells (Table S10). The identical lead SNP, rs9274663, was identified in both cases (Figure S3). These colocalization findings imply the potential presence of shared biological mechanisms between these immune cells and breast cancer, necessitating further investigation.

## 4. Discussion

Drawing from a substantial quantity of genomic data accessible to the public, we used immune cells to assess their causal role in gynecological tumors, the first Mendelian randomization analysis to explore the relationship between immune cells and gynecological cancers.

Based on our study, we found that increased CD11c^+^ HLA-DR^++^ cDC was associated with an increased risk of breast cancer. In contrast, HLA-DR plasmacytoid DC, and HLA-DR on DC were a risk factor for breast cancer and the results of the reverse Mendelian randomization analysis were not significant. Importantly, multivariable Mendelian analyses identified immunophenotypes with independent effects on breast cancer, which may indicate that CD11c^+^ HLA-DR^++^ cDC may have a more significant influence on breast cancer.

Breast cancer has emerged as a new global health concern since it is currently the most frequent cancer in the world and the primary cause of cancer-related deaths among women globally, according to recent statistics^39^. A poor prognosis can arise from immune evasion of breast cancer cells due to various immune factors that participate in immunosuppressive co-stimulation of receptors, including cytotoxic T lymphocyte-associated protein (CTLA)-4, programmed cell death-1 receptor (PD-1)^40^, and infiltration of suppressive immune cells (e.g., regulatory T cells (Tregs))^41^. A crucial modulator of antigen presentation, HLA-DR is an MHC class II protein expressed in monocytes from various people^42^. Elevated levels of monocytes have been linked to the advancement of breast cancer in previous research, and Qian et al. discovered that CCL2 can promote the metastasis of breast tumors by recruiting CD11 inflammatory monocytes^43^. Further research has revealed that monocyte CD11 molecules can stimulate the expression of CCL2 and IL-6. These two cytokines are most common in the tumor microenvironment, which can contribute to the growth and spread of tumors^44^. Cytokines influence the recruitment of inflammatory circulating cells, and these cells secrete additional substances that facilitate the growth and survival of cancer.

In our investigation, we found a negative correlation between the development of breast cancer and HLA-DR on plasmacytoid DC. Dendritic cells stimulate T cell-mediated immunity against cancer and play a role in establishing and maintaining peripheral and central immunological tolerance^45^. MHC-II molecules, which mature DCs express, provide antigens to T-lymphocytes, enabling T-cell differentiation into distinct effector T-cell subsets with varying roles. Similarly, mature DC's release of IL-12 can trigger T cells with particular anti-tumor immune responses^46^.

Moreover, it is interesting that lower levels of CD39^+^ resting Treg AC and CD28^+^ CD45RA^-^ CD8^+^ T cells are linked to the development of ovarian and cervical malignancies. There are numerous and intricate ways in which immune cells contribute to the development of breast cancer. A distinct subset of T cells known as Tregs is essential for mediating immunological tolerance^47^. The primary and rate-limiting ectonucleotides in charge of producing adenosine is CD39, expressed by T cells. Adenosine undergoes DC recruitment early in the immunological response, which sets off a particular immune response^48^. According to Maria et al., T cells expressing CD8^+^ undergo differentiation into effector cells that express NK receptors, stop expressing CD28, and undergo programmed cell death. Increased concentrations of CD28^+^ CD45RA^-^ CD8^+^ T cells in the peripheral blood of women may have an impact on the progression of cervical cancer^49^.

In this study, we used peripheral blood immune cell profiles as exposure factors to explore their causal effects on gynaecological tumors, thus providing important implications for enhancing clinical diagnosis and patient prognosis. All the genetic variables were obtained from GWAS, and 8926 SNP were used as genetic tools to explain their causal effects in gynaecological tumors. This study was based on IVW methodology and strict quality control, and multivariable Mendelian randomization was used to assess the effects of immune risk factors in a comprehensive MR causal inference analysis, so our results have high reliability.

The immunophenotypes have shown a certain degree of association with breast cancer, a result consistent with existing literature^50^. This association may further impact the field of gynecologic tumors, providing new research directions for immune modulation-related therapies and disease prognosis. However, further research is needed to fully understand the potential impact of immune phenotypes on gynecologic tumor health.

The existing study shows a phase I/II clinical trial evaluating the efficacy of 1 mg/kg of nivolumab alone and 1 mg/kg of nivolumab plus 3 mg/kg of ipilimumab in advanced breast cancer patients. Additionally, another trial is assessing single-agent PD-L1 inhibitor therapy in advanced breast cancer patients^51^. These studies will deepen our understanding of the role of immune phenotypes in breast cancer, providing crucial information for the clinical efficacy and immune relevance in breast cancer. This will help shape our future research directions, particularly in utilizing immune phenotypes to predict disease progression and outcomes in breast cancer patients, thereby advancing personalized medicine.

There are limitations to our investigation. First, only gynecologic tumor was analyzed as an outcome variable in this study, and some immune factors are associated with malignancy, not necessarily with cysts. Second, the study was based on a European database, which may not necessarily be extended to Asian populations. Third, we used a looser threshold to assess outcomes, which may increase some false positives. Finally, clinical trials need to be completed to validate the reliability of the results.

## 5. Conclusion

In conclusion, we demonstrated a causal relationship between several immune phenotypes and gynaecological tumors, especially with breast cancer, by comprehensive MR analysis, predicting that they might act as possible disease factors. Significantly after multivariable Mendelian randomization analysis, genetically indicated elevated level of CD11c^+^ HLA-DR^++^ cDC was still associated with a high risk of breast cancer. The relative counts of circulating immune cells may serve as a potential biomarker for predicting tumorigenesis, providing researchers with a novel avenue to explore early intervention and treatment of gynecological malignancies.

## Supplementary Material

Supplementary figures.

Supplementary tables.

## Abbreviations

AC: absolute cell
BRCA: breast cancer
CAR: chimeric antigen receptor
cDC: conventional dendritic cell
CTLA: cytotoxic T lymphocyte-associated protein
DC: dendritic cell
GWAS: genome-wide association study
IV: instrumental variable
IVW: inverse-variance weighted method
LASSO: least absolute selection and shrinkage operator
MAF: moderate allele frequency
MFI: median fluorescence intensities
MP: morphological parameters
MR: Mendelian randomization
MVMR: multivariable Mendelian randomization
OR: odds ratio
PD-1: programmed cell death-1 receptor
PP: posterior probabilities
RC: relative cell
SNP: single-nucleotide polymorphism
STROBE-MR: strengthening the reporting of observational studies in epidemiology-Mendelian randomization
VDR: vitamin D receptor
