# Supplementary material for: Causal Relationship between Immune Cells and Gynecological Cancers through Bidirectional and Multivariable Mendelian Randomization Analyses
Source: J Cancer. 2024 Feb 4;15(7):1880–9. doi: 10.7150/jca.92627 (PMC10905409; doi:10.7150/jca.92627)
Supplement: Supplementary file 1 — Supplementary figures. [file jcav15p1880s1.pdf]

Supplemental Figures

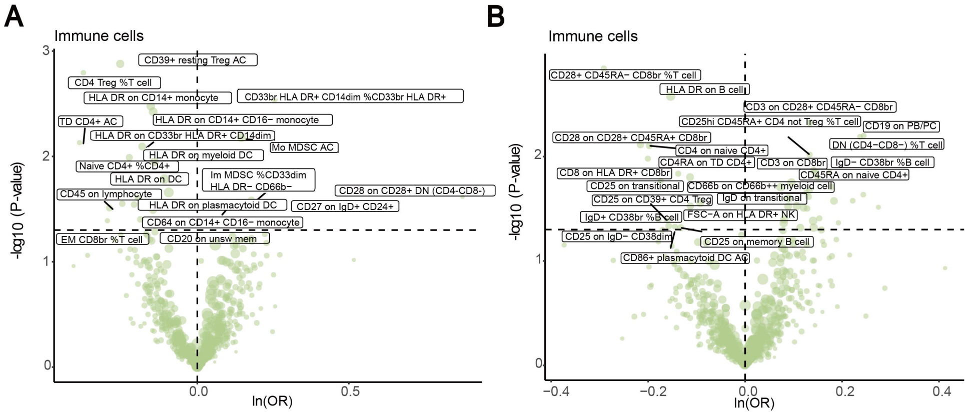

Fig. S1 (A) Illustration of the relationship between 731 immunophenotypes and ovary cancer, (B) cervix cancer.

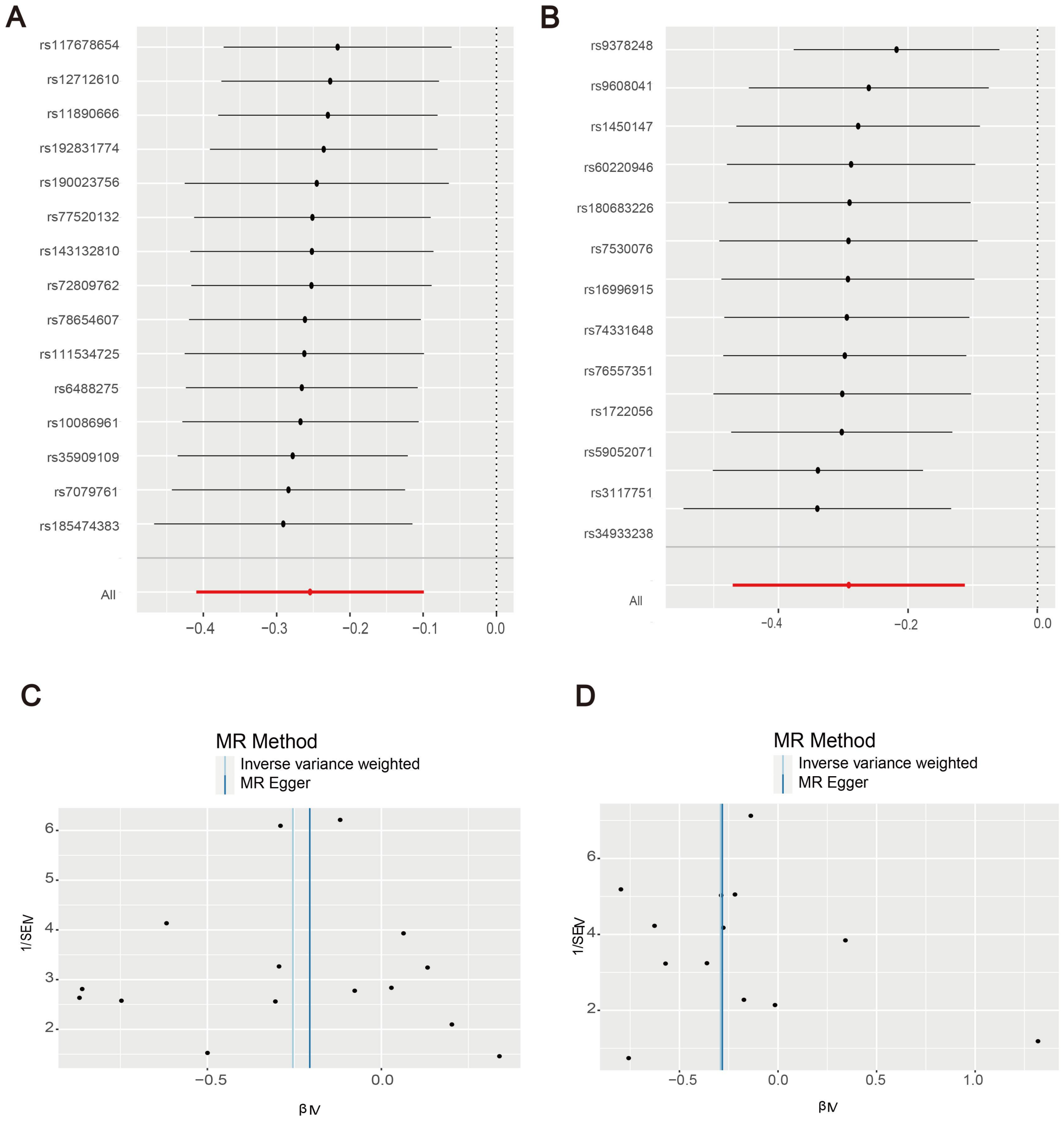

Fig. S2 (A) Forest diagram, (C) funnel plot showed the link between CD39^{+} resting Treg AC and ovary cancer. (B)forest diagram, (D) funnel plot showed CD28^{+} CD45RA^{-} CD8 T cell was negatively associated with cervical cancer.

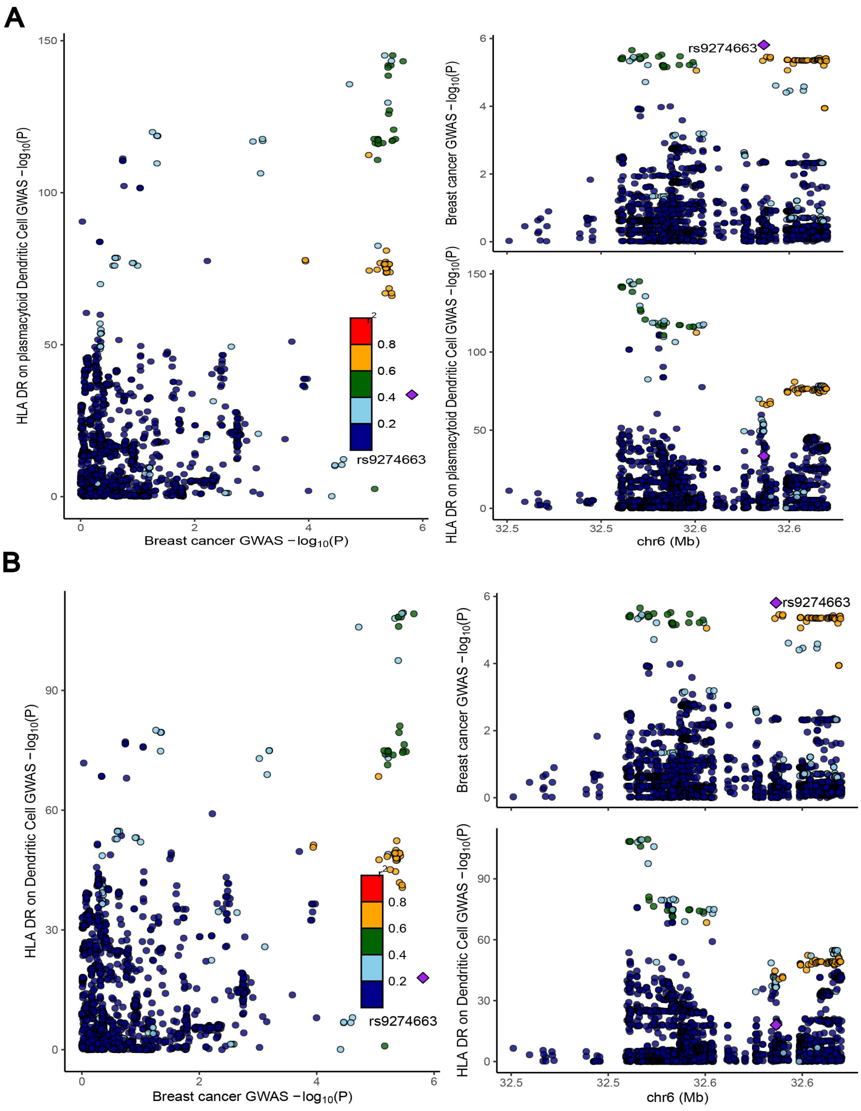

Fig. S3 Colocalization analysis of immune traits and breast cancer. The risk variation is designated and uniquely colored purple, while the other variants are colored according to their r2 value. Polymorphisms of single nucleotides, or SNPs (single-nucleotide polymorphisms). Among the top signals and in the upper right corner is the lead SNP, rs9274663, in the HLA-DR and breast cancer GWAS.

(A) HLA-DR plasmacytoid dendritic cell GWAS and breast cancer were significantly colocalized.

(B) Locus zoom showed the HLA-DR on dendritic cells and breast cancer were in diagonal lines.
